# DXA-Derived Visceral and Subcutaneous Adipose Tissue and Postmenopausal Breast Cancer Mortality

**DOI:** 10.3390/curroncol33020119

**Published:** 2026-02-17

**Authors:** Jennifer W. Bea, Shelby G. Ziller, Dylan Decker, Denise J. Roe, Andrew O. Odegaard, Heather M. Ochs-Balcom, Sarah M. Lima, Bette Caan, Jean Wactawski-Wende, Margaret S. Pichardo, Holly Harris, Zhao Chen

**Affiliations:** 1 Health Promotion Sciences, Mel and Enid Zuckerman College of Public Health, University of Arizona, Tucson, AZ 85724, USA; 2 Department of Epidemiology and Biostatistics, Mel and Enid Zuckerman College of Public Health, University of Arizona, Tucson, AZ 85724, USA; 3 Department of Psychology, University of Montana, Missoula, MT 59812, USA; 4 Department of Epidemiology and Biostatistics, University of California, Irvine, CA 92617, USA; 5 Department of Epidemiology and Environmental Health, State University of New York at Buffalo, Buffalo, NY 14214, USA; 6 Lombardi Comprehensive Cancer Center, Georgetown University, Washington, DC 20007, USA; 7 Kaiser Permanente Division of Research, Oakland, CA 94588, USA; 8 Department of Surgery, Hospital of the University of Pennsylvania, Penn Medicine, Philadelphia, PA 19104, USA; 9 Department of Epidemiology, Public Health Sciences Division, Fred Hutch Cancer Center, Seattle, WA 98109, USA

**Keywords:** breast cancer, mortality, adiposity, visceral adipose tissue

## Abstract

Background: Elevated abdominal adipose tissue at time of diagnosis is associated with breast cancer mortality. We sought to understand the association between abdominal adipose tissue (subcutaneous, SAT and visceral, VAT) assessed via dual-energy X-ray absorptiometry (DXA) and breast cancer mortality in the prevention setting. Methods: Women enrolled in the Women’s Health Initiative study with baseline whole-body DXA scans were included in the study (*n* = 9767). Causes of death were adjudicated up to 27 years of follow-up. Competing risk models were used to examine independent associations between baseline VAT, SAT, per 100 cm^2^, and breast cancer-specific deaths; findings were reported as sub-hazard ratios (SHR) and confidence intervals (CI). Time-varying analyses additionally included DXA at years 3 and 6. Covariates included demographic, lifestyle, and tumor factors. Results: Baseline VAT and SAT ranged from undetectable to 616.25 cm^2^ and 55.26–952.46 cm^2^, respectively. There were 738 incident breast cancer cases post-enrollment, and 87 breast cancer-related deaths. Median age at diagnosis was 62 years. In adjusted models, higher baseline VAT and SAT were significantly associated with higher risk breast cancer mortality (49% and 40%, respectively); time-varying models were similar. Conclusions: Higher VAT and SAT were similarly associated with breast cancer mortality in this group of postmenopausal women.

## Simple Summary

Prior research shows that higher levels of body fat, particularly deep belly fat that hugs the organs, can increase the risk of postmenopausal breast cancer. After a breast cancer diagnosis, belly fat has also been implicated in death. However, it is unclear if belly fat increases the risk of dying from breast cancer among women that have never been diagnosed with cancer before. This study showed that greater amounts of fat in two different fat compartments in the belly (i.e., surface subcutaneous and deep visceral) were equally associated with death from breast cancer. Future studies are needed to determine if these detailed measurements of belly fat are necessary for prediction of death from breast cancer or if simple measures like BMI and waist circumference are sufficient.

## 1. Introduction

In the United States (US), 42,170 women are expected to die from breast cancer in 2025 [1]. Postmenopausal breast cancer is an obesity-related cancer that is expected to rise in parallel with increasing obesity rates [2,3]. Despite progress in screening, detection, and treatment substantially improving breast cancer survivorship in the United States, breast cancer remains the second leading cause of cancer death for women [1,4].

Body mass index (BMI) has limitations as a metric for defining obesity, particularly among postmenopausal women [5,6]. We have shown that nearly the full range of BMI values is represented in each dual-energy X-ray absorptiometry (DXA)-derived quintile of total body fat (%) among postmenopausal women participating in the Women’s Health Initiative (WHI; e.g., BMI range 14.4–66.0 kg/m^2^ within Quintile 1 of %TBF and 18.9–69.1 kg/m^2^ in Quintile 5) [7]. Further, postmenopausal breast cancer risk has been positively associated with greater DXA-derived total body fat, even among women with normal BMI [8,9].

Advancements in DXA-based body composition software have only recently allowed for the measurement of adipose depots beyond total body and trunk, to include abdominal visceral adipose tissue (VAT) and subcutaneous adipose tissue (SAT). However, these advancements have not been fully leveraged in an epidemiological cohort due to limited longitudinal data. The WHI, with its historical DXA scans and 27 years of adjudicated breast cancer cases and mortality follow-up data, provides an ideal setting to address the relationship between abdominal adiposity and breast cancer mortality. We recently validated DXA measures of VAT and SAT in the WHI using the Apex 4.0 software and MRI data [10,11,12,13,14,15], and demonstrated an increased risk of incident breast cancer with greater abdominal adiposity, particularly VAT [10]. However, the relation between deaths due to breast cancer using detailed, DXA-derived adipose depots in the prevention setting has yet to be examined.

The present study is responsive to the call for more research to evaluate the prospective relation between body composition and cancer mortality earlier in the cancer continuum [16], and particularly site-specific mortality [17]. Our study addresses key gaps in the literature by examining the association between abdominal VAT, SAT, and breast cancer mortality for three key reasons: (1) Clinical relevance: abdominal body composition assessed by CT scans at diagnosis is increasingly used for predicting breast cancer survival [18]; (2) Accessibility: DXA is more widely available, less expensive, and involves lower radiation exposure than CT scans, making it more likely to be available in patient records prior to diagnosis [19]; and (3) Biological significance: VAT has been linked to greater metabolic dysfunction, inflammation, and immune dysregulation than other fat depots [20,21,22]. Dysfunction in these systems is included in the hallmarks of cancer [23].

We examined associations among participants that were cancer-free at enrollment for baseline abdominal VAT and SAT with breast cancer-specific mortality in a prospective cohort, while adjusting for demographics, behaviors, cancer stage, and disease subtype. We also assessed how time-varying levels of abdominal VAT and SAT were associated with breast cancer-specific mortality. Additionally, we examined breast cancer mortality limited to incident cases, while adjusting for breast cancer characteristics. We hypothesized that VAT would be a significant and independent driver of breast cancer mortality.

## 2. Materials and Methods

### 2.1. Study Population

This study was conducted with women in the WHI DXA cohort, which is nested in the WHI. The corresponding CONSORT diagram has been previously published [24]. In brief, a cohort of 11,450 women from the entire WHI sample (*N* = 161,808) was created from participants from the Tucson/Phoenix, AZ, Pittsburgh, PA, and Birmingham, AL clinical sites, regardless of clinical trial (CT) or observation study (OS) membership [10,24,25]. These participants received DXA scans at enrollment (1993–1998) and repeated scans at years 3 and 6.

Of the 11,450 participants in the DXA cohort, 579 had missing baseline scans, 145 had missing data on prevalent cancers, 731 had a previous history of cancer (except non-melanoma skin cancer), and 183 had unknown or missing mortality data. These participants were excluded to create a study population of 9767 women without cancer at WHI baseline who had complete cancer and mortality status during the 27 years of follow-up (28 February 2020). In secondary analyses the sample was limited to incident breast cancer cases with a DXA scan available within 4 years of diagnosis to create a study sample of 297 women.

### 2.2. Body Composition and Anthropometric Measurements

The primary exposure was abdominal adiposity, specifically VAT, SAT, and total abdominal adipose tissue (TAT) in the abdominal region of interest. These measures’ collection, calculation, and validation have been previously described [10]. In short, DXA scans (QDR2000, 2000+, or 4500W DXA models and QDR software v12.1, Hologic Inc., Bedford, MA, USA) were performed to measure total and regional adiposity and lean soft tissue at baseline, Year 3, and Year 6 [10]. A rigorous quality control program was employed across sites [26]. These historic scans were recently assessed centrally, with new software (APEX 4.0) and standardized procedures, to estimate VAT, SAT, and TAT [10]. The region of interest used was a 5 cm high section across the full width of the abdomen, approximately aligned with the fourth lumbar vertebra [10]. Using proprietary Hologic algorithms, VAT, SAT, and TAT were then quantified as area (cm^2^) within the 5 cm region of interest. Body weight and height were measured during a clinic visit using a balance beam scale and wall-mounted stadiometer, to the nearest 0.1 kg and 0.1 cm respectively. BMI was calculated by dividing total body mass (kg) by height squared (m^2^). Skeletal muscle index (SMI) was computed by dividing appendicular lean soft tissue (kg) by height squared (m^2^).

### 2.3. Outcome

Incident breast cancer diagnosis information in the DXA cohort has been previously described [10,25,27]. Cancer outcomes and deaths were self-reported at least annually by telephone, questionnaire, or in-person visits [10,25]. Breast cancers were confirmed if pathology reports substantiated a primary invasive or in situ cancer [10,25]. Adjudicated breast cancer was classified as ductal carcinoma in situ or invasive breast cancer [10,25]. Cancer-related hospitalizations, procedures, and treatments were investigated, with pathology reports and discharge summaries used for coding inpatient and outpatient diagnoses and cause of death [10,25]. Breast cancer characteristics, including age at diagnosis and stage of disease, were collected from these reports [10,25]. Breast cancer cases could be identified at death but could not be deemed a case solely based on cause of death [10,25]. Local adjudicators coded cancer sites and cause of death using ICD-O-2 codes [10,25].

Cancer mortality details have been comprehensively documented [7,25,28,29]. If participants were unreachable, listed contacts were contacted. Death certificates were obtained from next of kin or state vital statistics agencies. Cause of death was determined from death certificates, medical records, and autopsy reports. When other records were unavailable, only death certificates were used. Deaths were categorized as due to breast cancer, other cancers, or non-cancer causes. According to the WHI variable definitions, all breast cancer deaths were considered invasive. Time-to-death was measured from WHI enrollment to death or censored at the last follow-up for surviving participants. Those women lost to follow-up were searched via the National Death Index regularly.

### 2.4. Covariates

Socioeconomic, clinical, and behavioral characteristics were included in the analysis *a priori* to align with similar analyses in the literature [10]. Most demographic and clinical characteristics were reported at baseline and included age, race and ethnicity, education, income, alcohol consumption, family history of breast cancer, oral contraceptive (OC) use, hormone therapy (HT) use, metformin use, NSAID use, age at menarche, age at menopause, surgical menopause, and breastfeeding history. Age at first birth was included as a covariate and coded as never-had-a-term pregnancy, term pregnancy without live birth (referent), age under 20 years, 20–29 years, ≥30 years. Medication usage was defined as ever vs. never at baseline, except for HT use, which was defined as current, former, and never at baseline. Self-reported hysterectomy was used to define surgical menopause. Alcohol consumption was categorized as never, past, minimal (<1 drink/week), moderate (1 to <7 drinks/week), and heavy (>7 drinks/week) intake. Using self-report questionnaires, smoking status was included at baseline and years 1, 3, and 6. Physical activity was assessed annually through questionnaires, and metabolic equivalent values (MET-hr/wk) were then computed [30,31,32]. Physical function was assessed using the validated and self-reported RAND 36-Item Health survey and was reported based on the SF-36 Score from 0 to 100 with a higher score indicating more favorable health state [7]. Validated food frequency questionnaires were used to assess diet quality at baseline and additionally in Year 3 for OS participants. In addition, the Healthy Eating Index (HEI-2015) was calculated [33]. The behavioral measures for physical activity, diet, and smoking status were used at time points aligned with DXA scans in time-varying analysis. For variables that were measured more than once, only baseline measurements were used in baseline analyses. Cancer characteristics, including age at diagnosis and stage of disease, were taken from ICD-O-2 codes from adjudicated medical history files. Membership in the observational study, or the hormone therapy (HT), calcium and vitamin D (CaD), and dietary modification (DM) clinical trial arms were also included as covariates.

### 2.5. Statistical Analysis

Descriptive statistics were computed by breast cancer mortality status (deaths due to breast cancer versus the rest of the cohort) using *t*-tests, chi-square tests, or the appropriate non-parametric tests as indicated. Additional descriptive statistics for this cohort have been previously published [10,24].

Fine and Gray’s proportional sub-hazard model (i.e., competing risk regression) was used to assess the associations between the DXA-derived adiposity measures and breast cancer mortality. Models met proportional hazard assumptions and were deemed appropriate. In accordance with this method, three outcome variables were created for each analysis. For the primary baseline and time-varying analyses, the outcome variables were: (1) survived to last follow-up (censored), (2) breast cancer-related death (event), and (3) other cause of death (competing risk). Time from WHI enrollment to outcome was used as the underlying time metric.

Three models were fit for each primary exposure. Model 1, a multivariable-adjusted model, included height at baseline to account for overall body size and all other covariates listed above, Model 2 included Model 1 covariates (excluding height) plus baseline BMI, and Model 3 included Model 1 covariates plus baseline SMI; these models examined the potential for confounding by body size and skeletal muscle. There are concerns for collinearity as the variance inflation factors for BMI (>5) and SMI (>4) were notably high across all models. Therefore, models adjusted for baseline BMI and SMI are not presented as the main findings and are included for comparison to the literature. No significant interactions were identified between age and either VAT or SAT; therefore, interaction terms were not included in the final models. To align with the breast cancer mortality literature from the WHI, and known interactions in the literature at large, [7,8,34,35,36,37] we decided, *a priori,* to stratify analyses by age and BMI categories. We were underpowered to stratify by race and ethnicity. Additionally, Model 1 was used in time-varying adiposity models, which were developed using baseline, Year 3, and Year 6 DXA measurements with covariates updated accordingly by available time points.

The secondary analyses evaluated the association between breast cancer survival and the nearest pre-diagnosis measurements of DXA-derived adiposity. The sample for these analyses comprised participants with an incident breast cancer as their first cancer, DXA scan within one to four years of diagnosis, complete information on cause of death, and available breast cancer stage data (*n* = 297). Available DXA scans within one to four years of diagnosis were evaluated to address reverse causation. DXA scans within less than one year of diagnosis were not used for this analysis to control for variability of body composition. For these secondary analyses, competing risk regression outcomes were defined as: (1) survival to last follow-up (censored), (2) death from breast cancer (event), and (3) death due to other causes (competing risks). For this analysis, follow-up time was calculated from the date of breast cancer diagnosis. For each primary exposure in the secondary analyses, models were adjusted sequentially: unadjusted (Model 1); adjusted for age at diagnosis (Model 2); partially adjusted for age at diagnosis and breast cancer stage (Model 3), and fully adjusted for age at diagnosis, stage, income, smoking status, and hormone therapy use (Model 4).

Missing covariate data were addressed using multiple imputation via chained equations (MICE) with predictive mean matching for variables with limited value ranges. In the baseline analyses, the imputed variables included education, income, race/ethnicity, baseline height, alcohol intake, smoking status, physical activity (MET-hrs/week), physical function (SF-36 score), total energy intake (kcal/day), Health Eating Index-2015 (HEI-2015) score, baseline HT use, family history of breast cancer, age at first birth, age at menarche, total breastfeeding duration, age at menopause, and history of surgical menopause. For time-varying analyses, the abdominal adipose measurements at Years 3 and 6 were imputed if missing.

All analyses were performed using Stata 18 (StataCorp, College Station, TX, USA) and SAS 9.4 (SAS Institute Inc., Cary, NC, USA). A two-sided significance level of 0.05 was applied to all statistical tests.

## 3. Results

**Figure 1:**
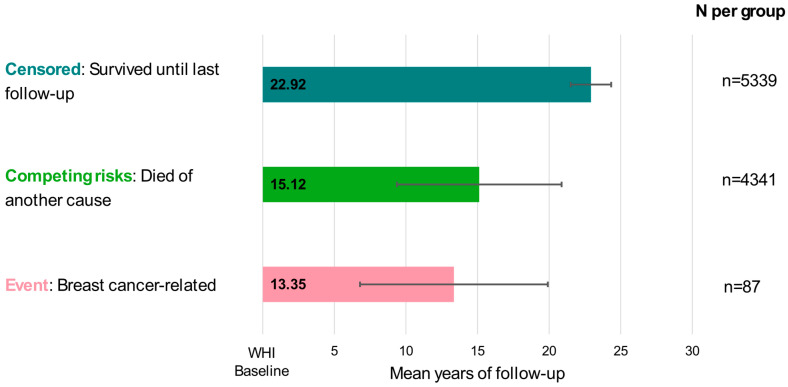
Mean years of follow-up for Fine and Gray’s competing risks regression model components in participants of the Women’s Health Initiative dual-energy X-ray absorptiometry cohort (*n* = 9767).

A summary of descriptive statistics by breast cancer mortality status is found in Table 1. A total of 9767 women were included in the analyses, representing a total of 189,179 person-years of follow-up. The median age at WHI baseline was 62 years (interquartile range [IQR]: 55–68), and the median BMI was 28.4 kg/m^2^ (IQR: 25.1–31.7). Baseline VAT and SAT ranged from undetectable (zero) to 616.25 cm^2^ and 55.26–952.46 cm^2^, respectively. For those who developed an incident breast cancer as their first cancer, the mean age at breast cancer diagnosis was 73.5 ± 9.1 years and the mean age at death for those who died from that breast cancer was 77.8 ± 8.3 years. Mean years of follow-up for those who died of breast cancer was 13.4 ± 6.6 (Figure 1).

Compared to women who did not die of breast cancer, women who died from breast cancer had substantially higher baseline VAT (mean ± SD: 194.4 ± 86.5 cm^2^ vs. 166.2 ± 81.73 cm^2^, *p* < 0.001) and SAT (mean ± SD: 442.79 ± 150.9 cm^2^ vs. 380.3 ± 137.9 cm^2^, *p* < 0.001). Furthermore, women who died from breast cancer had lower alcohol consumption and were more likely to have a first-degree female relative with breast cancer. Of those women who died from breast cancer, their breast cancer was primarily estrogen receptor positive (ER+) (36.78%), in the regional or distant stages (45.98%), had a solid tumor greater than 2 cm (56.32%), and had positive lymph nodes (56.32%). Characteristics and distribution of cases according to adipose quartiles are illustrated in Table 2. The distribution of cases and adiposity over BMI categories is shown in Supplemental Table S1.

**Figure 2:**
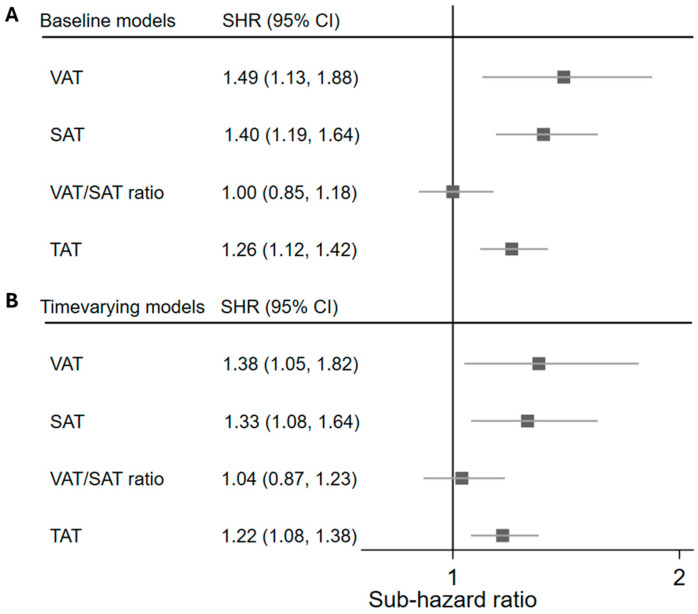
Multivariable-adjusted associations between (**A**) baseline and (**B**) time-varying adiposity variables and breast cancer-related mortality in the Women’s Health Initiative dual-energy X-ray absorptiometry cohort (*n* = 9767; breast cancer-related deaths: 87). VAT, SAT, and TAT per 100 cm^2^ are limited to the new 5 cm high abdominal region of interest. Multivariable models adjusted for: age at baseline, region, education, income, race and ethnicity, hormone therapy trial arm, diet modification trial arm, calcium and vitamin D trial arm, height at baseline, alcohol intake, smoking status, physical activity (MET-hrs/wk), physical function (SF 36 score), total energy intake (kcal/day), HEI-2015 score, hormone therapy, aspirin, metformin, female relative with breast cancer, age at menarche, age at first birth, total number of months of breastfeeding, age at menopause, and surgical menopause. VAT: Visceral adipose tissue; SAT: Abdominal subcutaneous adipose tissue; TAT: Total abdominal adipose tissue; Q: Quartile; SHR: Sub-hazard ratio; CI: Confidence interval; WHI: Women’s Health Initiative; DXA: Dual-energy X-ray absorptiometry.

In fully adjusted baseline models, higher VAT, SAT, and TAT (per 100 cm^2^) were associated with a higher risk of breast cancer-related death; SHR (95% CI): 1.49 (1.13–1.97), 1.40 (1.19–1.64), and 1.26 (1.12–1.42), respectively (Figure 2). Quartile 4 of SAT [3.46 (1.75–6.83)] and TAT [2.73 (1.39–5.36)] was associated with more breast cancer-related deaths compared to Quartile 1 (Supplemental Figure S1). Time-varying models incorporating body composition measurements at baseline, Year 3, and Year 6 further supported these findings. The significant associations between continuous VAT, SAT, and TAT with breast cancer mortality persisted in fully adjusted time-varying models. In quartile models, the relationship with Quartile 4 of SAT was attenuated, but the significant association with Quartile 4 of TAT persisted. VAT/SAT ratio was not significantly associated with breast cancer deaths in any models.

In sensitivity analysis, body size was accounted for by separately adjusting for BMI (Model 2) and SMI (Model 3). The association between VAT and breast cancer mortality was attenuated by adding BMI or SMI across continuous and quartile models (Table 3). However, the significant associations with breast cancer mortality and continuous SAT and TAT, as well as SAT Quartile 4 remained; the association with Quartile 4 of TAT was attenuated.

Associations were further analyzed through an *a priori* decision to examine stratified models by age and BMI categories (Table 4). In women aged 60–69, there was a significantly increased risk of breast cancer mortality with increased VAT, SAT, and TAT (per 100 cm^2^); these associations were not significant in the other age groups. In BMI stratified models, women with obesity demonstrated a significantly increased risk of breast cancer mortality with higher VAT, SAT, and TAT (per 100 cm^2^), while overweight associations were limited to SAT and TAT (per 100 cm^2^). There were no significant associations between abdominal adipose depots and breast cancer mortality among women with normal weight, as categorized by BMI.

In secondary analyses, restricted to women with a first incident breast cancer diagnosis and DXA scan captured within one to four years prior to diagnosis, women who died from breast cancer were more likely to have had regional or distant disease (*n* = 297; Supplemental Table S2). There were no demographic differences. Mean years between DXA scans and diagnosis were 2.3 ± 1.0 for those alive at last follow-up, 2.2 ± 1.00 for competing risks, and 1.8 ± 0.9 for breast cancer-related deaths. In competing risks models, higher pre-diagnosis abdominal adiposity was not associated with an increased risk of breast cancer death (Table 5). In this subgroup, DXA-measured abdominal adiposity obtained 1–4 years prior to diagnosis did not predict subsequent breast cancer mortality.

## 4. Discussion

Abdominal adipose has garnered significant attention for its prognostic value among breast cancer patients [18]. However, our study is the first to examine the association between abdominal adipose depots and breast cancer mortality in a prospective cohort with nearly 30 years of adjudicated follow-up. In this setting, our data do *not* support a superior or independent association between VAT and breast cancer deaths *compared with* SAT and TAT, as hypothesized. Higher baseline abdominal VAT, SAT, and TAT were significantly associated with increased breast cancer mortality and the VAT/SAT ratio association with breast cancer mortality was null. Additionally, both baseline and time-varying models supported these results. To try and address the longer time periods between the scans and deaths, we conducted our secondary analyses with participants with scans within 1 to 4 years of death. Though there were no significant findings, the null results may reflect an insufficient event count (*n* = 297; deaths = 24) rather than a lack of association, justifying further investigation. As illustrated in our previous work, the majority of TAT consists of SAT leading to high correlations (Spearman’s rank correlation: 0.97, *p*-value < 0.001) [24]. This is similarly seen in this study and is illustrated in the similar findings between TAT and SAT throughout all the analyses.

Though our study was prospective and not limited to scans at diagnosis, as in the majority of studies in the literature, our finding that SAT is associated with breast cancer-related deaths is in alignment with the non-significant increase in the risk of breast cancer-specific mortality with higher SAT shown in the B-SCANS study, a non-metastatic breast cancer study with CT at the time of diagnosis (*n* = 3139) [18,38]. These findings are contrary to our *a priori* hypothesis and to prior emphasis on VAT as the fat depot considered most harmful. In a prospective study within the WHI, we found that breast cancer incidence was more highly associated with DXA-derived VAT [10], which is what led us to hypothesize that VAT may be more important in the prevention setting with average-risk postmenopausal women. Adipose tissue acts as the only appreciable source of estrogen postmenopause, and thus estrogen and adiposity have a complex interaction that influences adipose distribution and function. Nationally, ER+ breast cancer consists of around 70% of all breast cancers, and was also the most common among our cases [39]. As indicated in the literature, body composition and menopausal status may be better predictors of initiation and promotion from ER+ breast cancer rather than mortality or incidence of other subtypes [40].

Biologically, VAT is hypothesized to be the greater driver for obesity-related chronic diseases, like breast cancer, given it is the more hormonally active tissue [18,38]. VAT has been linked to metabolic dysfunction, inflammation, and immune dysfunction, which are considered hallmarks of cancer [23]. However, there are pathways in which SAT can also drive obesity-related chronic diseases [38]. For example, adipose tissue macrophages are the key producers of proinflammatory cytokines, which create the hallmark low-grade inflammation seen in obesity [41]. The macrophage accumulation is more pronounced in VAT, but is also seen in SAT [42]. In addition, SAT contributes to systemic adipokine secretion and metabolic dysfunction and has been shown to correlate more strongly with breast adipose tissue than VAT, providing several potential pathways through which SAT could influence breast tumor behavior and progression [38,43,44]. Therefore, SAT may also influence breast tumor promotion and cancer progression through similar pathways [38]. Additionally, abdominal SAT has been shown to have a higher correlation to breast adipose tissue than VAT, and may be, in part, influencing the associations [43,44].

Taken together, these findings suggest that there are mechanistic differences between the abdominal depot contributions to mortality versus initiation of postmenopausal breast cancer. The similar magnitudes of association for VAT and SAT, together with the null findings for the VAT/SAT ratio suggest that the overall burden of abdominal adiposity may be more important for postmenopausal breast cancer-related death than the relative distribution of VAT and SAT. When both depots are positively associated with risk and moderately to strongly correlated, the ratio can mask their individual contribution, making a null association more plausible. However, it is important to note that though the cohort itself was relatively large, the number of breast cancer deaths was very small (*n* = 87) especially compared to the number of cases (*n* = 738). This reduces the reliability of these estimates, and further research in a sample with a greater event rate is warranted to test potential effect differences between VAT and SAT.

Of note, there were some differences between VAT and SAT across stratified analyses, such as increased risk of breast cancer mortality with higher VAT among women aged 60–69 and women with obesity. However, the limited number of deaths and wide confidence intervals across strata underscore the need for larger studies to replicate these findings. Stratified analyses, in particular, should be interpreted with caution, as they may be unstable and have increased risk of Type I and II error.

In this study, we further examined the contribution of SMI to the associations (Model 3) because excess adiposity and sarcopenia (low muscle mass and strength) have been independently associated with increased breast cancer-specific and overall mortality [18]. We found that SMI attenuated the abdominal adipose associations with breast cancer-related deaths. One way to enhance SMI is through exercise, and National guidelines recommend exercise in cancer survivorship settings [45,46,47], due to improved quality of life, cancer control, and physical function, as well as reduced treatment toxicity, among other benefits [48,49,50]. Importantly, most survivors do not require physician approval or monitoring [46]. While it is not possible to separate the beneficial effects of exercise and improved skeletal muscle mass on breast cancer outcomes, the data support the promotion of exercise as a public health strategy that addresses both muscle preservation and adipose reduction in breast cancer prevention and control.

One may wonder about the influence of weight loss in the breast cancer survivorship setting as well, but unfortunately it is beyond the scope of this study, and data related to weight loss interventions in breast cancer survivors are limited [48]. Current clinical guidelines noted insufficient evidence to make recommendations about loss in this setting. Thus, the results of pending trials are of great interest, including the Breast Cancer Weight Loss (BWEL) trial—a randomized clinical trial on weight loss among overweight women with stage II or III breast cancer—and the TeleHealth Resistance Exercise Intervention to Preserve Dose Intensity and Vitality in Elder Breast Cancer Patients (THRIVE-65)—a trial on exercise, protein intake, and body composition during chemotherapy [51,52].

### Strengths and Limitations

Our ability to characterize and adjust for numerous potential confounders in competing risk models was a strength, as well as the availability of repeated measures and follow-up time that increase the findings’ reliability. Competing risks models account for other causes of death of breast cancer survivors and mitigate potential collider bias. However, there were some limitations in this study. Results cannot be applied to premenopausal or male breast cancer, and we were not able to stratify by disease stage, treatment, or recurrence due to the limited number of deaths. Importantly, breast cancer-specific mortality is not influenced by biological risk factors alone, but also by social determinants of health (SDOH). Some SDOH covariates, such as education and income, were included in our models, but not all 12 SDOH variables were available [53]. The small number of breast cancer-related deaths may lead to over-fitting of the covariate-adjusted models and limited statistical power to detect association in age- and BMI-stratified analyses, resulting in wide confidence intervals. Additionally, residual confounding from overall body size may still influence the models. In the secondary analyses, the small sample size and deaths (*n* = 24) limited power further, and, due to WHI protocol, progression, treatment, and care access covariates were unavailable. In addition, potentially significant changes in body composition and metabolic health may have occurred between baseline and pre-diagnosis scans (e.g., weight gain, age-related loss of lean mass), which may have diluted associations between pre-diagnosis adiposity and mortality and contributed to null findings.

DXA scans were completed during the postmenopausal period only and do not represent adiposity across the life course, nor do they reflect body composition of breast cancer cases close to time of death. For some women, the baseline DXA assessment occurred 20 or more years before breast cancer diagnosis. Therefore, our findings could be better interpreted as reflecting earlier life or long-term adiposity rather than adiposity immediately preceding diagnosis. Pre-diagnosis DXA scans obtained 1–4 years before diagnosis may also be influenced by preclinical disease-related changes (e.g., cancer-related weight or muscle loss), further limiting their ability to predict mortality. Nevertheless, the WHI DXA cohort remains one of the largest and longest follow-ups of participants with repeated DXA scans and adjudicated breast cancer outcomes and was best positioned to answer the call for earlier analyses of body composition associations with breast cancer mortality.

While the DXA-derived VAT and SAT measures were validated against MRI [10,11,12,13,14,15], and DXA has the advantage of being more accessible compared to the gold standard imaging techniques of CT and MRI, direct comparison between DXA and CT or MRI measures is not currently advisable. DXA is two-dimensional and CT and MRI are three-dimensional in nature. Thus, a future, well-powered, head-to-head comparison of harmonized anthropometric and body composition data is needed to identify the optimal metric for prediction of breast cancer mortality risk.

## 5. Conclusions

Abdominal subcompartments of VAT and SAT were both significantly associated with higher risk of breast cancer-related death among postmenopausal women. Our data demonstrated that individual assessment of VAT and SAT may not be necessary in mortality assessment, but rather that TAT is sufficient. Future well-powered studies are needed to determine whether there are differences between demographic groups that would enable prevention efforts to be targeted to those most likely to benefit.

## Figures and Tables

**Table 1:** Baseline demographic characteristics of women who died from breast cancer and did not die from breast cancer among women of the Women’s Health Initiative dual-energy X-ray absorptiometry cohort (mean ± SD or *n* (column %), as appropriate).

Variable	Died from Breast Cancer (*n* = 87)	Did not Die from Breast Cancer (*n* = 9680)	*p*
**Age at baseline (y)**	63.28 ± 6.71	63.20 ± 7.39	0.93
**Ethnicity: Hispanic or Latina**	<10	662 (6.84%)	0.65
**Race**			
American Indian or Alaska Native	<10	147 (1.52%)	0.14
Asian or Pacific Islander *	<10	37 (0.38%)	1.00
Black	13 (14.94%)	1368 (14.13%)	0.78
White	67 (77.01%)	7824 (80.83%)	0.53
**Education**			0.80
Less than high school	<10	870 (8.99%)	
High school or GED completed	19 (21.84%)	2213 (22.86%)	
Vocational training, technical school or some college	37 (42.53%)	3619 (37.39%)	
College degree	10 (11.49%)	794 (8.20%)	
Some post-graduate or professional school	<10	807 (8.34%)	
Graduate degree	<10	1316 (13.60%)	
**Income**			0.76
Less than $20,000	26 (29.89%)	2409 (24.89%)	
$20,000 to $34,999	23 (26.44%)	2519 (26.02%)	
$35,000 to $49,999	15 (17.24%)	1687 (17.43%)	
$50,000 to $74,999	11 (12.64%)	1365 (14.10%)	
$75,000 and greater	<10	982 (10.14%)	
**Physical activity (MET-hrs/wk)**	9.95 ± 13.03	11.41 ± 13.76	0.27
**Physical function (RAND 36 score)**	77.18 ± 19.98	78.60 ± 21.29	0.54
**Total energy intake (kcal/day)**	1746.11 ± 872.69	1656.24 ± 801.52	0.30
**HEI-2015 score**	63.42 ± 9.05	63.46 ± 10.68	0.97
**Smoking Status**			0.60
Never	52 (59.77%)	5257 (54.31%)	
Former	28 (32.18%)	3531 (36.48%)	
Current	<10	763 (7.88%)	
**Alcohol intake**			0.02
Non-drinker	18 (20.69%)	1636 (16.90%)	
Past drinker	28 (32.18%)	2098 (21.67%)	
Minimal (<1 drink per week)	25 (28.74%)	3125 (32.28%)	
Moderate (1 to <7 drinks per week)	12 (13.79%)	1979 (20.44%)	
Heavy (7+ drinks per week)	<10	762 (7.87%)	
**Age at Menarche (y)**			0.32
10 or less	<10	565 (5.84%)	
11–12	36 (41.38%)	3865 (39.93%)	
13–14	33 (37.93%)	4098 (42.33%)	
15+	<10	1114 (11.51%)	
**Age at first birth (y)**			0.16
never pregnant	<10	824 (8.51%)	
none	<10	240 (2.48%)	
<20	10 (11.49%)	1630 (16.84%)	
20–29	57 (65.52%)	5494 (56.76%)	
30+	<10	582 (6.01%)	
**Age at menopause**	47.87 ± 6.37	47.52 ± 6.80	0.65
**Total number of months of breastfeeding**			0.80
Never breastfed	38 (43.68%)	4265 (44.06%)	
1–6 months	25 (28.74%)	2664 (27.52%)	
7–12 months	11 (12.64%)	1141 (11.79%)	
13–23 months	<10	846 (8.74%)	
24+ months	<10	637 (6.58%)	
**Surgical Menopause**			0.95
Surgical menopause	33 (37.93%)	3330 (34.40%)	
Natural menopause	46 (52.87%)	5381 (55.59%)	
**Hormone therapy use**			0.29
Never	37 (42.53%)	4604 (47.56%)	
Former	19 (21.84%)	1524 (15.74%)	
Current	31 (35.63%)	3547 (36.64%)	
**Oral contraceptive use**	30 (34.48%)	3612 (37.31%)	0.59
**NSAID use**	50 (57.47%)	5792 (59.83%)	0.65
**Metformin use**	<10	60 (0.62%)	0.53
**Family history of cancer**			
Female relative with cancer	46 (52.87%)	4341 (44.85%)	0.19
Female relative with breast cancer	22 (25.29%)	1545 (15.96%)	0.03
Male relative with cancer	24 (27.59%)	3233 (33.40%)	0.24
**Anthropometry**			
**BMI (kg/m^2^)**	30.78 ± 7.02	28.18 ± 5.69	<0.01
**Waist Circumference (cm)**	91.31 ± 15.37	85.81 ± 13.11	<0.01
**BMI category**			<0.01
Underweight (<18.5)	<10	73 (0.75%)	
Normal (18.5–24.9)	20 (22.99%)	3090 (31.92%)	
Overweight (25.0–29.9)	29 (33.33%)	3403 (35.15%)	
Obesity I (30.0–34.9)	15 (17.24%)	1927 (19.91%)	
Obesity II (35.0–39.9)	14 (16.09%)	783 (8.09%)	
Obesity III (≥40)	<10	380 (3.93%)	
**DXA Body Composition**			
**VAT (100 cm^2^)**	1.94 ± 8.65	1.66 ± 8.17	<0.01
**SAT (100 cm^2^)**	4.43 ± 1.51	3.80 ± 1.38	<0.01
**TAT (100 cm^2^)**	6.37 ± 2.23	5.47 ± 2.07	<0.01
**VAT to SAT ratio**	0.43 ± 0.12	0.43 ± 0.14	0.72
**Total body fat (%)**	45.84 ± 7.12	43.86 ± 7.28	0.01
**Total body fat (kg)**	36.76 ± 12.63	32.57 ± 11.47	<0.01
**Skeletal muscle index (kg/m^2^)**	6.02 ± 1.32	5.65 ± 0.98	0.01
**Cancer Characteristics among breast cancer deaths (*n* = 87)**			
**Stage**			
Local	24 (27.57%)		
Regional or distant	40 (45.98%)		
**Tumor Characteristics**			
ER positive	32 (36.78%)		
ER negative	24 (27.57%)		
Triple Negative	12 (13.79%)		
**Tumor Size**			
Less than 1 cm	<10		
1 to 2 cm	12 (13.79%)		
Greater than 2 cm	49 (56.32%)		
**Positive lymph node**	49 (56.32%)		

* Asian and Pacific Islander group is not reported in detail by case status due to small cell sizes. In the total sample there were 2 Asian Indian, 11 Chinese, 4 Filipino, 6 Japanese, 1 Korean, and 13 Other Asian women; 83 women identified as more than one race; totals for all race categories will exceed 100% because participants reported all races with which they identified. For the new 5 cm high abdominal region of interest: VAT: Visceral adipose tissue, SAT: Abdominal subcutaneous adipose tissue, TAT: Total abdominal adipose tissue; *n*: Number; SD: Standard Deviation; BMI: Body mass index; MET-hrs/wk: metabolic equivalent task hours per week; HEI: Healthy Eating Index 2015; WHI: Women’s Health Initiative; DXA: Dual-energy X-ray absorptiometry, ER: Estrogen receptor.

**Table 2:** Characteristics and descriptives of adiposity measure quartiles in participants of the Women’s Health Initiative dual-energy X-ray absorptiometry cohort (*n* = 9767).

	Quartiles			
	Q1	Q2	Q3	Q4
**VAT (100 cm^2^)**				
BCa mortality cases/N	15/2441	16/2442	26/2442	30/2442
Range	0.00–105.94	105.95–158.80	158.80–217.15	217.19–616.25
Mean (SD)	70.32 (±24.93)	132.43 (±15.00)	186.37 (±16.51)	276.80 (±51.75)
**SAT (100 cm^2^)**				
BCa mortality cases/N	12/2441	18/2442	18/2442	39/2442
Range	55.26–283.48	283.53–368.99	369.01–465.14	465.17–952.46
Mean (SD)	217.09 (±48.36)	326.36 (±24.39)	413.54 (±27.51)	566.25 (±86.00)
**VAT/SAT ratio**				
BCa mortality cases/N	24/2441	16/2442	25/2442	22/2442
Range	0.00–0.33	0.33–0.42	0.42–0.51	0.51–3.33
Mean (SD)	0.27 (±0.05)	0.38 (±0.02)	0.46 (±0.03)	0.61 (±0.10)
**TAT (100 cm^2^)**				
BCa mortality cases/N	11/2441	20/2442	20/2442	36/2442
Range	74.31–397.73	397.75–535.19	535.24–686.95	687.01–1227.98
Mean (SD)	292.75 (±74.88)	466.79 (±39.19)	606.58 (±43.31)	823.05 (±106.63)

For the new 5 cm high abdominal region of interest: VAT: Visceral adipose tissue; SAT: Abdominal subcutaneous adipose tissue; TAT: Total abdominal adipose tissue; BCa: Breast cancer; N: Number; SD: Standard Deviation; Q: Quartile; WHI: Women’s Health Initiative; DXA: Dual-energy X-ray absorptiometry.

**Table 3:** Sensitivity analysis of the association of baseline abdominal body composition and breast cancer mortality with additional adjustment for BMI (Model 2) and skeletal muscle index (Model 3), using competing risks time-to-event models in the Women’s Health Initiative dual-energy X-ray absorptiometry cohort (*n* = 9767; BCa-related deaths = 87).

Variable	Cases/Person-Years	Model 1 SHR (95% CI)	Model 2 SHR (95% CI)	Model 3 SHR (95% CI)
**VAT (100 cm^2^)**		**1.49 (1.13, 1.97)**	1.25 (0.83, 1.88)	1.31 (0.93, 1.83)
**VAT quartiles (Q1 ref.)**	15/48,715.07			
VAT Q2	16/48,332.63	1.16 (0.58, 2.34)	1.12 (0.53, 2.38)	1.08 (0.54, 2.19)
VAT Q3	26/47,059.82	1.67 (0.86, 3.24)	1.56 (0.68, 3.59)	1.41 (0.71, 2.81)
VAT Q4	30/45,071.43	2.03 (1.00, 4.11)	1.43 (0.55, 3.76)	1.45 (0.67, 3.14)
**SAT (100 cm^2^)**		**1.40 (1.19, 1.64)**	**1.44 (1.12, 1.86)**	**1.34 (1.11, 1.62)**
**SAT quartiles (Q1 ref.)**	12/47,198.93			
SAT Q2	18/47,568.93	1.48 (0.72, 3.05)	1.69 (0.77, 3.72)	1.44 (0.69, 2.98)
SAT Q3	18/47,398.98	1.49 (0.70, 3.19)	2.17 (0.83, 5.70)	1.35 (0.61, 3.00)
SAT Q4	39/47,012.10	**3.46 (1.75, 6.83)**	**5.19 (1.85, 14.56)**	**2.92 (1.38, 6.18)**
**VAT/SAT ratio**		1.00 (0.85, 1.18)	0.94 (0.78, 1.13)	0.93 (0.78, 1.11)
**VAT/SAT ratio quartiles (Q1 ref.)**	24/50,096.68			
VAT/SAT ratio Q2	16/48,208.42	0.69 (0.37, 1.28)	0.62 (0.33, 1.18)	0.64 (0.34, 1.18)
VAT/SAT ratio Q3	25/46,712.38	1.13 (0.63, 2.04)	1.03 (0.55, 1.90)	0.98 (0.54, 1.77)
VAT/SAT ratio Q4	22/44,161.46	0.82 (0.41, 1.66)	0.67 (0.32, 1.40)	0.64 (0.31, 1.32)
**TAT (100 cm^2^)**		**1.26 (1.12, 1.42)**	**1.32 (1.07, 1.64)**	**1.22 (1.06, 1.42)**
**TAT quartiles (Q1 ref.)**	11/47,719.11			
TAT Q2	20/48,091.41	1.41 (0.71, 2.82)	1.52 (0.77, 3.01)	1.28 (0.63, 2.60)
TAT Q3	20/47,153.85	1.61 (0.79, 3.26)	2.02 (0.84, 4.84)	1.49 (0.71, 3.11)
TAT Q4	36/46,214.57	**2.73 (1.39, 5.36)**	2.75 (0.94, 8.02)	**2.15 (1.00, 4.62)**

VAT, SAT, and TAT are limited to the new 5 cm high abdominal region of interest. Model 1 adjusted for: age at baseline, region, education, income, race and ethnicity, hormone therapy trial arm, diet modification trial arm, calcium and vitamin D trial arm, height at baseline, alcohol intake, smoking status, physical activity (MET-hrs/wk), physical function (SF 36 score), total energy intake (kcal/day), HEI-2015 score, hormone therapy, aspirin, metformin, female relative with breast cancer, age at menarche, age at first birth, total number of months of breastfeeding, age at menopause, and surgical menopause. Model 2 adjusted for: Model 1 and body mass index; Model 3 adjusted for: Model 1 and skeletal muscle mass index. Model 1 findings from Figure 1 and Supplemental Figure S1 have been reproduced here for ease of comparison. Bolding indicates significant findings. VAT: Visceral adipose tissue; SAT: Abdominal subcutaneous adipose tissue; TAT: Total abdominal adipose tissue; Q: Quartile; SHR: Sub-hazard ratio; CI: Confidence interval; WHI: Women’s Health Initiative; DXA: Dual-energy X-ray absorptiometry.

**Table 4:** Multivariable-adjusted associations between baseline adiposity variables and BCa-related mortality stratified by age at baseline and baseline BMI category in the Women’s Health Initiative dual-energy X-ray absorptiometry cohort (*n* = 9767; BCa-related deaths: 87).

	Cases/ Person-Years	VAT (100 cm^2^)	SAT (100 cm^2^)	VAT/SAT Ratio	TAT (100 cm^2^)
Stratification		SHR (95% CI)	SHR (95% CI)	SHR (95% CI)	SHR (95% CI)
Age at Baseline ^a^					
50–59	25/72,405.48	1.76 (0.93–3.33)	1.24 (0.96–1.59)	1.11 (0.97–1.26)	1.22 (0.97–1.52)
60–69	43/82,652.77	**1.47 (1.02–2.12)**	**1.53 (1.21–1.93)**	0.93 (0.74–1.16)	**1.32 (1.11–1.56)**
70–79	19/34,120.69	1.18 (0.66–2.12)	1.28 (0.92–1.80)	0.92 (0.62–1.37)	1.14 (0.91–1.45)
Baseline BMI ^b^					
Normal weight	20/61,154.04	1.68 (0.49–5.82)	1.55 (0.73–3.31)	0.95 (0.65–1.40)	1.32 (0.77–2.23)
Overweight	29/66,809.88	1.14 (0.59–2.21)	**2.01 (1.25–3.25)**	0.82 (0.59–1.15)	**1.44 (1.05–1.99)**
Obese	38/59,471.02	**1.86 (1.19–2.90)**	**1.51 (1.11–2.05)**	1.07 (0.81–1.40)	**1.51 (1.19–1.93)**

VAT, SAT, and TAT are limited to the new 5 cm high abdominal region of interest. a: Model adjusted for: age at baseline, income, hormone therapy trial arm, diet modification trial arm, calcium and vitamin D trial arm, smoking status, physical activity (MET-hrs/wk), physical function (SF 36 score), hormone therapy, metformin, age at menarche, age at menopause; b: Model adjusted for: age at baseline, region, income, race and ethnicity, hormone therapy trial arm, diet modification trial arm, calcium and vitamin D trial arm, alcohol intake, smoking status, physical activity (MET-hrs/wk), physical function (SF 36 score), total energy intake (kcal/day), HEI-2015 score, hormone therapy, aspirin, metformin, female relative with breast cancer, age at menarche, age at first birth, total number of months of breastfeeding, age at menopause, surgical menopause. Bolding indicates significant findings. VAT: Visceral adipose tissue; SAT: Abdominal subcutaneous adipose tissue; TAT: Total abdominal adipose tissue; Q: Quartile; SHR: Sub-hazard ratio; CI: Confidence interval; WHI: Women’s Health Initiative; DXA: Dual-energy X-ray absorptiometry.

**Table 5:** Competing risks time-to-event models: association of abdominal body composition (nearest pre-diagnosis DXA within one to four years) with breast cancer mortality in incident cases in the Women’s Health Initiative dual-energy X-ray absorptiometry cohort.

	BCa Deaths Among BCa Cases (*n* = 297; BCa-Related Deaths = 24)				BCa Deaths Among Only Invasive BCa Cases (*n* = 236; BCa-Related Deaths = 23)
Variable	Model 1 SHR (95% CI)	Model 2 SHR (95% CI)	Model 3 SHR (95% CI)	Model 4 SHR (95% CI)	Model 4 SHR (95% CI)
**VAT (100 cm^2^)**	1.01 (0.62, 1.64)	1.01 (0.62, 1.63)	1.00 (0.61, 1.64)	0.97 (0.59, 1.60)	1.06 (0.65, 1.73)
**SAT (100 cm^2^)**	1.11 (0.84, 1.48)	1.11 (0.84, 1.49)	1.16 (0.84, 1.60)	1.16 (0.83, 1.64)	1.21 (0.84, 1.73)
**VAT/SAT ratio**	0.84 (0.64, 1.11)	0.84 (0.64, 1.11)	0.79 (0.60, 1.04)	0.81 (0.60, 1.10)	0.84 (0.62, 1.15)
**TAT (100 cm^2^)**	1.05 (0.86, 1.28)	1.05 (0.86, 1.28)	1.07 (0.86, 1.33)	1.06 (0.85, 1.33)	1.10 (0.87, 1.39)

VAT, SAT, and TAT are limited to the new 5 cm high abdominal region of interest. *n* = 297 women observed for 3745 person-years total in BCa deaths among BCa cases. *n* = 236 women observed for 2923.62 person-years in BCa deaths among invasive BCa case. DXA scans were within one to four years of diagnosis. Competing risks are other causes of death. Model 4 used MICE to address missing covariates. Model 1: unadjusted; Model 2: adjusted for age at diagnosis; Model 3: adjusted for age at diagnosis and cancer stage; Model 4: adjusted for age at diagnosis, cancer stage, income, smoking status, and hormone therapy. VAT: Visceral adipose tissue; SAT: Abdominal subcutaneous adipose tissue; TAT: Total abdominal adipose tissue; SHR: Sub-hazard ratio; CI: Confidence interval; WHI: Women’s Health Initiative; DXA: Dual-energy X-ray absorptiometry.

## Data Availability

Restrictions apply to the availability of these data. Data were obtained from the Women’s Health Initiative (WHI) and are available at whi.org for all researchers who apply through the papers and proposals committee.

## Supplementary Materials

The following are available online at https://www.mdpi.com/article/10.3390/curroncol33020119/s1, Table S1: Average VAT area, SAT area, and VAT/SAT ratio stratified by baseline BMI category and breast cancer mortality status; Table S2: Baseline demographic characteristics of women with incident breast cancer with DXA scan within 4 years of diagnosis stratified by those who died from breast cancer and those who did not die from breast cancer (*n*= 297; mean ± SD or *n* (column %), as appropriate). Figure S1: Multivariable-adjusted associations between A) baseline and B) time varying adiposity variables and breast cancer related mortality in the Women’s Health Initiative dual-energy X-ray absorptiometry cohort (*n* = 9767; BCa-related deaths: 87).

## Abbreviations

The following abbreviations are used in this manuscript:

WHI	Women’s Health Initiative
DXA	Dual-energy X-ray absorptiometry
BMI	Body mass index
VAT	Visceral adipose tissue
SAT	Abdominal subcutaneous adipose tissue
TAT	Total abdominal adipose tissue
CT	Clinical trial
OS	Observational study
OC	Oral contraceptive
HT	Hormone therapy
NSAID	Non-Steroidal Anti-Inflammatory Drug
MET	Metabolic equivalent values (hours/week)
HEI 2015	Healthy Eating Index 2015
CaD	Calcium and Vitamin D clinical trial
DM	Dietary modification clinical trial
MICE	Multiple imputations using chained equations
SHR	Sub-hazard ratios
CI	Confidence intervals
IQR	Interquartile range
BCa	Breast cancer
Q	Quartile
ER+	Estrogen receptor positive
