# Supplementary material for: DXA-Derived Visceral and Subcutaneous Adipose Tissue and Postmenopausal Breast Cancer Mortality
Source: Curr Oncol. 2026 Feb 17;33(2):119. doi: 10.3390/curroncol33020119 (PMC12939695; doi:10.3390/curroncol33020119)
Supplement: Supplementary file 1 [file curroncol-33-00119-s001.zip › Bea - Adiposity and BC - Supplemental table 1.pdf]

Supplemental Table S1: Average VAT area, SAT area, and VAT/SAT ratio stratified by baseline BMI category and breast cancer mortality status among women of the Women's Health Initiative dual-energy X-ray absorptiometry cohort (n=9,767)

		VAT area ($100cm^2$)		SAT area ($100cm^2$)		VAT/SAT ratio	
**BMI category at baseline**	**N**	**Mean**	**SD**	**Mean**	**SD**	**Mean**	**SD**
**Underweight (< 18.5)**	73	0.27	0.21	1.26.	37.81	0.19	0.11
BCa mortality cases	0	NA	NA	NA	NA	NA	NA
BCa mortality non-cases	73	0.27	0.21	1.26	0.38	0.19	0.11
**Normal (18.5 - 24.9)**	3110	0.95	0.43	2.52	0.68	0.36	0.12
BCa mortality cases	20	1.02	0.49	2.68	0.76	0.36	0.10
BCa mortality non-cases	3090	0.95	0.43	2.52	0.68	0.36	0.12
**Overweight (25.0 - 29.9)**	3432	1.64	0.50	3.73	0.68	0.44	0.12
BCa mortality cases	29	1.65	0.42	3.98	0.62	0.42	0.11
BCa mortality non-cases	3403	1.64	0.50	3.73	0.68	0.44	0.12
**Obesity I (30.0 - 34.9)**	1942	2.24	0.59	4.74	0.77	0.48	0.14
BCa mortality cases	15	2.47	0.60	4.88	0.61	0.51	0.13
BCa mortality non-cases	1927	2.24	0.59	4.74	0.77	0.48	0.14
**Obesity II (35.0 - 39.9)**	797	2.69	0.67	5.68	0.84	0.49	0.14
BCa mortality cases	14	2.64	0.44	5.65	0.76	0.47	0.09
BCa mortality non-cases	783	2.69	0.67	5.68	0.85	0.49	0.15
**Obesity III (≥ 40)**	389	2.90	0.81	6.75	0.96	0.44	0.16
BCa mortality cases	<10	2.98	0.95	7.13	0.74	0.43	0.16
BCa mortality non-cases	380	2.90	0.81	6.74	0.97	0.45	0.16

VAT, SAT, and TAT are limited to the new 5cm high abdominal region of interest. VAT: Visceral adipose tissue, SAT: Abdominal subcutaneous adipose tissue, TAT: Total abdominal adipose tissue, BCa: Breast cancer, N: Number, SD: Standard Deviation
