# Supplementary material for: DXA-Derived Visceral and Subcutaneous Adipose Tissue and Postmenopausal Breast Cancer Mortality
Source: Curr Oncol. 2026 Feb 17;33(2):119. doi: 10.3390/curroncol33020119 (PMC12939695; doi:10.3390/curroncol33020119)
Supplement: Supplementary file 1 [file curroncol-33-00119-s001.zip › Bea - Adiposity and BC - Supplemental table 2.pdf]

Supplemental Table S2: Baseline demographic characteristics of women with incident breast cancer with DXA scan within 4 years of diagnosis stratified by those who died from breast cancer and those who did not die from breast cancer among women of the Women's Health Initiative dual-energy X-ray absorptiometry cohort (N=297; mean ± SD or N (column %), as appropriate).

**Variable**	**Died from breast cancer (n=24)**	**Did not die from breast cancer (n=273)**	**p**
**Age at diagnosis (y)**	70.46 ± 7.52	70.85 ± 7.25	0.80
**Stage of BCa***			<0.001
Local	11 (45.83%)	212 (77.66%)	
Regional/Distant	13 (54.17%)	61 (22.34%)	
**Tumor characteristics**			
ER Positive	10 (41.67%)	201 (73.63%)	<0.001
ER Negative	11 (45.83%)	33 (12.09%)	<0.001
**Tumor Size**			
Less than 1cm	<10	80 (29.30%)	0.01
1 to 2 cm	<10	101 (37.00%)	0.24
Greater than 2 cm	17 (70.83%)	92 (33.70%)	<0.001
**Positive lymph nodes**	16 (66.67%)	114 (41.76%)	0.02
**Income**			0.34
Less than $20,000	<10	41 (15.02%)	
$20,000 to $34,999	<10	80 (29.30%)	
$35,000 to $49,999	<10	56 (20.51%)	
$50,000 to $74,999	<10	39 (14.29%)	
$75,000 and greater	<10	39 (14.29%)	
**Smoking Status**			0.38
Never	15 (62.50%)	142 (52.01%)	
Former	<10	105 (38.46%)	
Current	<10	23 (8.42%)	
**Hormone therapy use**			0.50
Never	10 (41.67%)	134 (49.08%)	
Former	<10	33 (12.09%)	
Current	<10	106 (38.83%)	
**Anthropometry**			
**BMI**	29.58 ± 6.14	29.54 ± 5.82	0.97
**Waist Circumference (cm)**	88.93 ± 15.31	89.03 ± 13.45	0.97
**BMI category**			0.88
Underweight (< 18.5)	<10	<10	
Normal (18.5 - 24.9)	<10	68 (24.91%)	
Overweight (25.0 - 29.9)	<10	90 (32.97%)	
Obesity I (30.0 - 34.9)	<10	65 (23.81%)	
Obesity II (35.0 - 39.9)	<10	29 (10.62%)	
Extreme Obesity III (≥ 40)	<10	19 (6.96%)	
**DXA Body Composition**			
VAT, cm^2	1.92 ± 9.23	1.91 ± 8.70	0.90
SAT, cm^2	4.35 ± 1.56	4.10 ± 1.42	0.41
TAT, cm^2	6.27 ± 2.37	6.00 ± 2.15	0.55
VAT to SAT ratio	0.41 ± 0.12	0.44 ± 0.14	0.32

Total body fat %	45.36 ± 6.51	44.78 ± 7.24	0.70
Total body fat (kg)	35.01 ± 11.85	35.07 ± 11.69	0.98
Skeletal muscle index (kg/height m^2)	5.84 ± 1.35	5.92 ± 0.98	0.78

*Local includes in situ. VAT, SAT, and TAT are limited to the new 5cm high abdominal region of interest. BCa: Breast cancer, VAT: Visceral adipose tissue, SAT: Abdominal subcutaneous adipose tissue, TAT: Total abdominal adipose tissue, ER: Estrogen receptor
