# Supplementary material for: DXA-Derived Visceral and Subcutaneous Adipose Tissue and Postmenopausal Breast Cancer Mortality
Source: Curr Oncol. 2026 Feb 17;33(2):119. doi: 10.3390/curroncol33020119 (PMC12939695; doi:10.3390/curroncol33020119)
Supplement: Supplementary file 1 [file curroncol-33-00119-s001.zip › Bes - Adiposity and BC - Supplemental Figure S1 Legend.pdf]

Supplemental Figure S1.

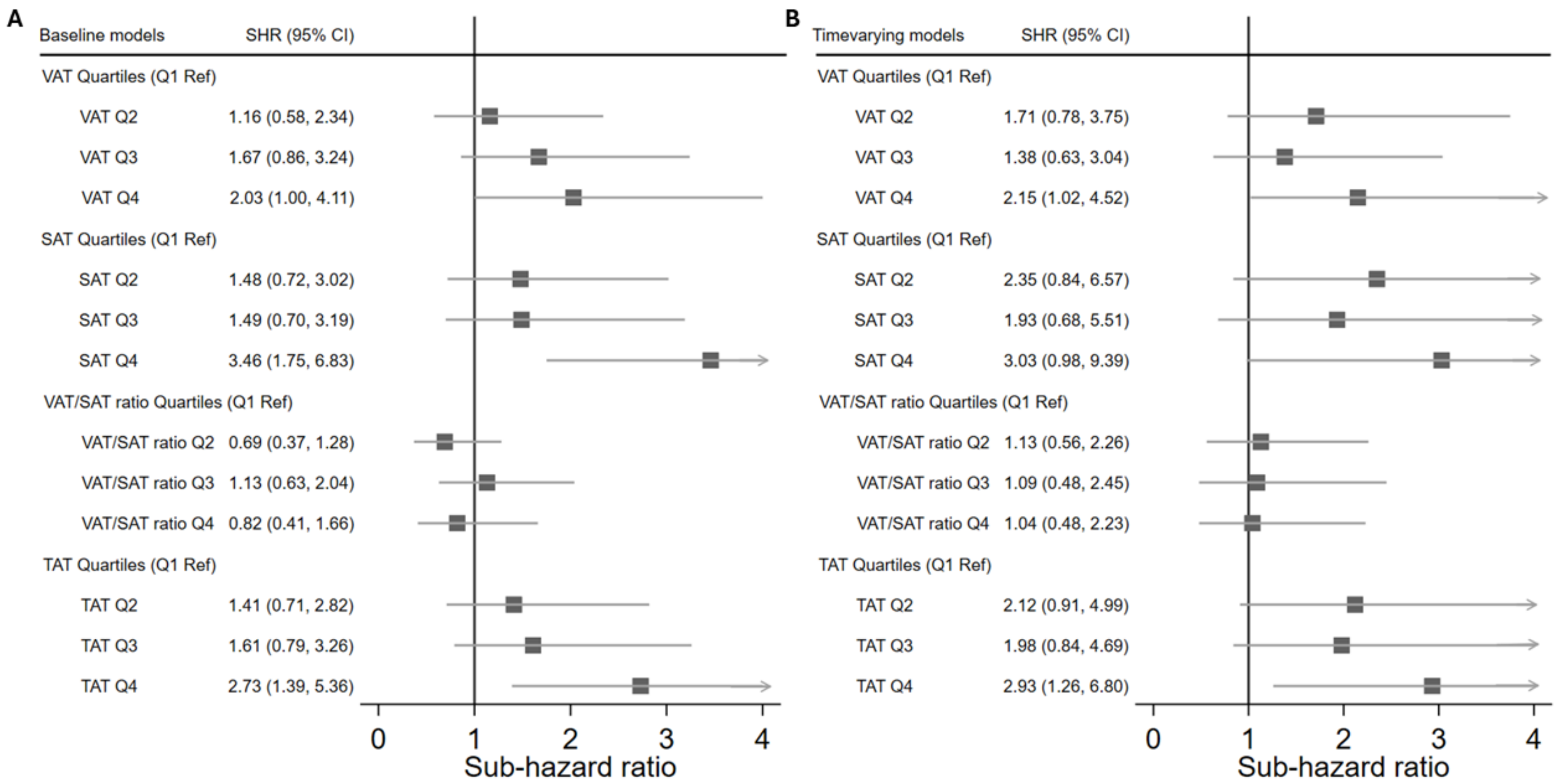

Supplemental Figure S1. Multivariable-adjusted associations between A) baseline and B) time varying adiposity variables and breast cancer related mortality in the Women's Health Initiative dual-energy X-ray absorptiometry cohort (n=9,767; BCa-related deaths: 87). VAT, SAT, and TAT are limited to the new 5cm high abdominal region of interest. Multivariable models adjusted for: age at baseline, region, education, income, race and ethnicity, hormone therapy trial arm, diet modification trial arm, calcium and vitamin D trial arm, height at baseline, alcohol in-take, smoking status, physical activity (MET-hrs/wk), Physical function (SF 36 score), total energy intake (kcal/day), HEI-2015 score, hormone therapy, aspirin, metformin, female relative with breast cancer, age at menarche, age at first birth, total number of months of breastfeeding, age at

menopause, and surgical menopause. VAT: Visceral adipose tissue, SAT: Abdominal subcutaneous adipose tissue, TAT: Total abdominal adipose tissue, Q: Quartile, SHR: Sub-hazard ratio, CI: Confidence interval, WHI: Women's Health Initiative, DXA: dual-energy X-ray absorptiometry.
