# Supplementary figures and images for: DXA-Derived Visceral and Subcutaneous Adipose Tissue and Postmenopausal Breast Cancer Mortality
Source: Curr Oncol. 2026 Feb 17;33(2):119. doi: 10.3390/curroncol33020119 (PMC12939695; doi:10.3390/curroncol33020119)

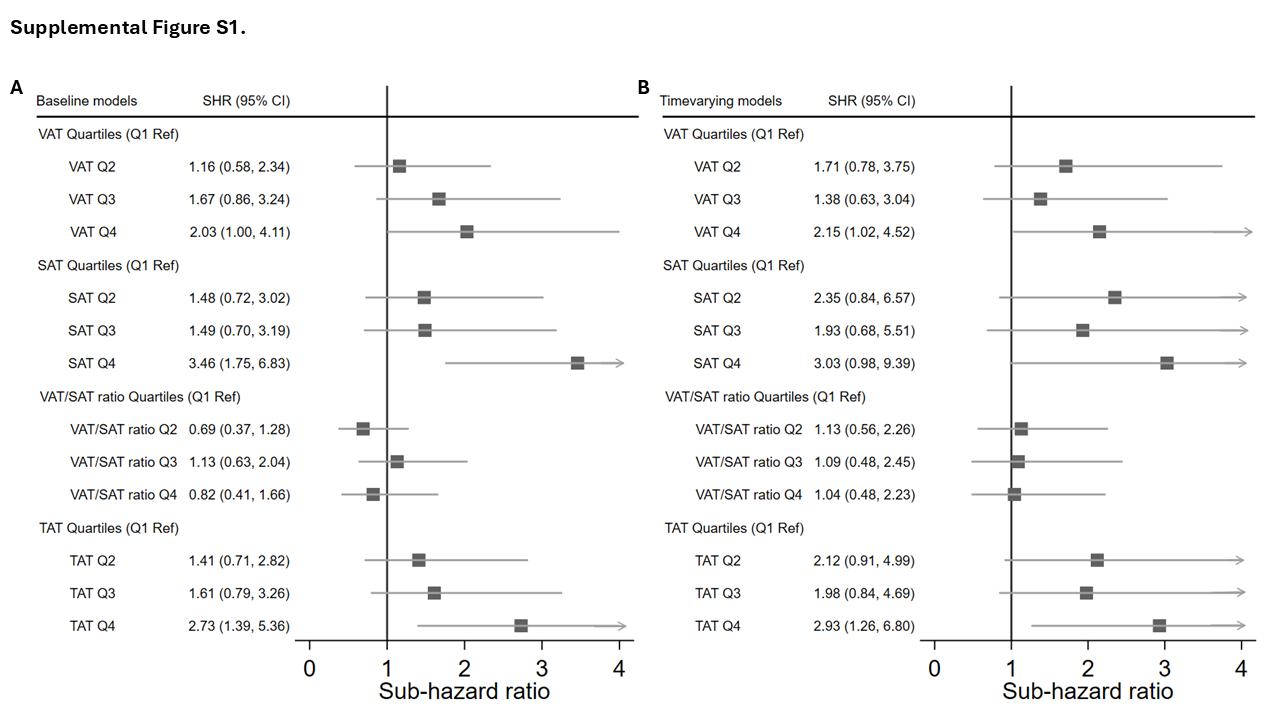

Supplement: Supplementary file 1 [file curroncol-33-00119-s001.zip › Bea - Adiposity and BC - Supplemental figure 1.png]
